# Supplementary material for: Complexome profiling of the Chlamydomonas psb28 mutant reveals TEF5 as an early PSII assembly factor
Source: Plant Cell. 2025 Mar 17;37(6):koaf055. doi: 10.1093/plcell/koaf055 (PMC12164590; doi:10.1093/plcell/koaf055)
Supplement: koaf055_Supplementary_Data [file koaf055_supplementary_data.zip › psb28_interactive.zip!/plots/intensity/qualityPlots/peptides/_Cre03.g154950.t1.2.html]

### Cre03.g154950.t1.2

Trivial name:   
SeqCoverage [median]: 9.00  
Mass: 40.43 kDa  
  
 

| Sequence | MissedCleavage | Mass [Da] | wtIntensity | muIntensity | Score |
| --- | --- | --- | --- | --- | --- |
| DLYHVPGIGR | 0 | 1125.59 | 198.14 | 0.00 | 53.33 |
| MLNSAFNALAK | 0 | 1178.61 | 660.91 | 0.00 | 76.68 |
| TIVNNESPEIMR | 0 | 1401.69 | 497.30 | 411.98 | 95.48 |
|  |  |  | 1356.3 | 412.0 |  |
